# Antimicrobial Resistance and the Spectrum of Pathogens in Dental and Oral-Maxillofacial Infections in Hospitals and Dental Practices in Germany

**DOI:** 10.3389/fmicb.2021.676108

**Published:** 2021-06-02

**Authors:** Annika Meinen, Annicka Reuss, Niklas Willrich, Marcel Feig, Ines Noll, Tim Eckmanns, Bilal Al-Nawas, Robby Markwart

**Affiliations:** ^1^Robert Koch Institute, Unit 37: Nosocomial Infections, Surveillance of Antimicrobial Resistance and Consumption, Berlin, Germany; ^2^Department of Oral and Maxillofacial Surgery, Plastic Surgery, University Medical Center of the Johannes Gutenberg-University Mainz, Mainz, Germany; ^3^Institute of General Practice and Family Medicine, Jena University Hospital, Jena, Germany

**Keywords:** antimicrobial resistance, AMR, surveillance, pathogen spectrum, odontogenic infections, dental care, AMR in oral-maxillofacial infections

## Abstract

Data on microbiological profiles in odontogenic infections are scarce. This study aimed to analyze the spectrum of pathogens and antimicrobial resistance in clinical isolates from dental and oral-maxillofacial clinical settings in Germany. We analyzed 20,645 clinical isolates (dental practices: *n* = 5,733; hospitals: *n* = 14,912) from patients with odontogenic infections using data (2012–2019) from the German *Antimicrobial-Resistance-Surveillance* (ARS) system. A total of 224 different species from 73 genera were found in clinical isolates from dental practices, and 329 different species from 97 genera were identified in isolates from hospital patients. In both hospitals and dental practices *Streptococcus* spp. (33 and 36%, respectively) and *Staphylococcus* spp. (21 and 12%, respectively) were the most frequently isolated microorganisms. In *Streptococcus* spp. isolates from hospitals, penicillin and aminopenicillin resistance proportions were 8.0% (95%CI 4.7–14.9%) and 6.9% (95%CI 4.7–9.9%), respectively. Substantially lower resistance proportions of penicillin and aminopenicillin were observed in dental practices [2.6% (95%CI 1.4–4.7%) and 2.1% (95%CI 1.1–4.0%), respectively]. Among *Staphylococcus aureus* isolates from hospital patients methicillin resistance proportions were 12.0% (95%CI 9.7–14.8%), which was higher than in isolates from dental practices (5.8% (95%CI 4.1–8.1%)]. High clindamycin and macrolide resistance proportions (>17%) were observed in *Streptococcus* spp. and *Staphylococcus aureus* isolates. In *Klebsiella* spp. isolates carbapenem resistance proportions were <1%. In sum, substantial antibiotic resistance was observed in isolates from odontogenic infections, which calls for strengthened efforts in antibiotic stewardship and infection prevention and control measures in both hospitals and dental practices.

## Introduction

The oral flora, alongside the gastrointestinal microbiome, is one of the most diverse accumulations of microorganisms in the human body. The most common pathogens in the oral cavity in healthy people include *Streptococcus* spp., *Granulicatella* spp., and *Veillonella* spp. (Aas et al., 2005; Dewhirst et al., 2010), which can also cause dental and oral-maxillofacial infections under certain conditions, such as caries, periodontitis, endodontic infections and tonsillitis (Scannapieco, 2013; Wade, 2013; Døving et al., 2020). In addition to these commensals, other bacterial pathogens are associated with infections of the oral cavity, such as *Staphylococcus* spp. (3) and *Candida* spp. (Singh et al., 2014). Recent evidence indicates that oral microorganisms are also responsible for systemic diseases (Han and Wang, 2013), such as cardiovascular diseases (Desvarieux et al., 2010; Koren et al., 2011; El Kholy et al., 2015). According to the World Health Organization (WHO), many of the bacterial microorganisms associated with dental and oral-maxillofacial infections are also associated with resistance to antibiotics (Tacconelli et al., 2018). Antibiotic-resistant bacteria are associated with a significant mortality and morbidity (Cassini et al., 2019) and therefore pose a severe health threat worldwide (WHO, 2014).

Data on the spectrum of pathogens and their antibiotic resistance profiles are available in detail for various infections types, such as urinary tract (Tandogdu and Wagenlehner, 2016; Klingeberg et al., 2018) and bloodstream infections (Weiner et al., 2016; CDC, 2019; Diekema et al., 2019; ECDC, 2019; Markwart et al., 2019). Despite the clinical relevance and frequency of dental and oral-maxillofacial infections, there is a lack of recent data on the spectrum of clinical pathogens and associated antimicrobial resistance for those infections, especially from multicenter studies. Such data are necessary for the development of clinical recommendations and guidelines on the treatment dental and oral-maxillofacial infections.

This study therefore aimed to analyze the microbiological profile (i.e., pathogen spectrum and antimicrobial resistance) of clinical isolates from dental and oral-maxillofacial medicine and to compare microbiological profiles between dental practices and hospitals in Germany.

## Materials and Methods

### Study Design, Data Source and Outcomes

We conducted a retrospective observational study on clinical isolates from dental and oral-maxillofacial medicine from 2012 to 2019, using data retrieved from the German Antimicrobial Resistance Surveillance (ARS) database (Noll et al., 2012). ARS is the national laboratory-based surveillance system for antimicrobial resistance in Germany and a priority area for the German Antimicrobial Resistance Strategy (DART). Laboratories that participate on a voluntary basis report data obtained from routine clinical microbiological testing of isolates from patients treated in hospitals and outpatient care clinics. In addition to results of pathogen identification and antimicrobial susceptibility testing, the ARS data include pseudonymized information on medical facilities such as care level, ward type and geographical location, patient characteristics such as age and gender, and type of specimen. High quality data are assured by checking the database for plausibility during data reporting and regularly validating the data for completeness and consistency.

The outcomes of this study were the (i) proportional distribution of pathogens identified in clinical isolates from dental and oral-maxillofacial clinical settings, stratified by setting (i.e., hospital and outpatient care), and (ii) the antibiotic resistance proportions among all tested clinical isolates of the most common pathogens.

### Selection of Isolates

In September 2020, we extracted data (2012–2019) on isolates from dental and oral-maxillofacial clinics (outpatient dental practices or hospitals) from the ARS database. We only included the patients’ first isolate per specimen per quarter in order to avoid including multiple isolates of one patient from one infection episode. Isolates from samples labeled as “screening” were excluded. Only isolates from the following specimen materials were included: Swabs (from abscesses, surgery, wound, tongue, not specified sites and other) and biopsies (from tissue, abscesses, joints, not specified sites, and other).

### Study Variables and Definitions

Care settings were categorized into hospital care and ambulatory dental practices. The regional origins of isolates were grouped into five major German regions: Northeast (federal states of Mecklenburg-West Pomerania, Brandenburg, Berlin, Saxony-Anhalt), Southeast (Bavaria, Saxony, Thuringia), Southwest (Hesse, Rhineland-Palatinate, Saarland, Baden-Wurttemberg), West (North Rhine-Westphalia) and Northwest (Lower Saxony, Hamburg, Schleswig-Holstein). Patients’ gender was categorized into female and male. Their ages were grouped into the following categories: 0–19, 20–39, 40–64, >65 years; and were also expressed as medians with interquartile ranges (IQRs).

In order to calculate the proportional distributions of pathogens identified in clinical isolates, we included all isolates and categorized them into the ten most common genera. Antimicrobial resistance profiles were analyzed for *Streptococcus* spp., *Staphylococcus aureus*, and *Klebsiella* spp. if the total number of tested isolates per antibiotic was greater than 100 in each care setting.

Based on recommendations from relevant clinical guidelines (Schöfer et al., 2011; Al-Nawas and Karbach, 2017), the following antibiotics were included in the analyses of antimicrobial profiles: Penicillin, aminopenicillins (amoxicillin, ampicillin), markers for methicillin resistance (oxacillin, flucloxacillin), second-generation cephalosporins (cefuroxim), third-generation cephalosporins (ceftriaxon, cefotaxim, ceftazidim), clindamycin, macrolides (erythromycin, clarithromycin), fluoroquinolones (moxifloxacin, levofloxacin), and carbapenems (imipenem, meropenem, ertapenem). An isolate was considered resistant against an antibiotic group if the susceptibility test results were classified by the laboratories as “resistant” for at least one antibiotic of the antibiotic group. In Germany, the majority of laboratories use the *European Committee on Antimicrobial Susceptibility Testing* (EUCAST) guidelines for interpreting susceptibility testing results (European Committee on Antimicrobial Susceptibility Testing, 2020).

### Statistical Analyses

All statistical analyses were performed using R version 3.6.1 (R Core Team, 2013) and the “survey” package (version 4.0) (Lumley, 2020). Estimates of antibiotic resistance proportions are expressed as percentages with 95% confidence intervals (95% CI) accounting for clustering at hospital / dental practice level using the survey package (Ayobami et al., 2020a, b). In order to study potential differences in antibiotic resistance in isolates from dental practices and hospitals as well as temporal changes in antibiotic resistances, multivariable logistic regression analyses were performed. The following predictors were included: Year of sampling, patient gender, patient age group, German region, and care type. All variables were treated as categorical, except year of sampling, which was treated as a continuous variable. The regression analyses also accounted for clustering at hospital / dental practice level (Lumley, 2020).

## Results

### Baseline Characteristics

In total, 20,645 clinical isolates from dental and oral-maxillofacial settings were included in the study. These isolates were obtained from 299 outpatient dental practices and 34 hospitals. The baseline characteristics of the analyzed isolates are outlined in Table 1. The majority (72.2%) of isolates were derived from patients treated in hospitals, while the remaining 27.8% were derived from dental practices. The isolates were collected from healthcare facilities in all German regions. In dental practices, the majority of clinical isolates were derived from female patients (female/male ratio = 1.14), while in hospitals, isolates were mainly collected from male patients (female/male ratio: 0.67). The majority of isolates were from middle aged (40–64 years) and elderly patients (>65 years).

**TABLE 1:** Baseline characteristics of clinical isolates from dental and oral-maxillofacial settings in Germany (2012–2019).

		*Dental practices*	*Hospitals*
*Total number of isolates (n, %)*		5733 (27.8%)	14,912 (72.2%)
*Patient gender*			
	Female (n, %)	2530 (44.1%)	4928 (33.0%)
	Male (n, %)	2215 (38.6%)	7342 (49.2%)
	Unknown (n, %)	988 (17.2%)	2642 (17.7%)
	Gender ratio (f/m)	1.14	0.67
*Age*			
	0–19 years (n, %)	358 (6.2%)	358 (2.4%)
	20–39 years (n, %)	996 (17.4%)	2591 (17.4%)
	40–64 years (n, %)	2090 (36.5%)	4549 (30.5%)
	>65 years (n, %)	2266 (39.5%)	7400 (49.6%)
	Age (median, IQR)	59 (41 – 73)	64 (46 – 81)
	NA (n, %)	23 (0.40%)	14 (0.094%)
*Year of sampling*			
	2012 (n, %)	314 (5.5%)	698 (4.7%)
	2013 (n, %)	369 (6.4%)	793 (5.3%)
	2014 (n, %)	357 (6.2%)	766 (5.1%)
	2015 (n, %)	615 (10.7%)	1917 (12.9%)
	2016 (n, %)	822 (14.3%)	2161 (14.5%)
	2017 (n, %)	901 (15.7%)	2881 (19.3%)
	2018 (n, %)	1105 (19.3%)	2959 (19.8%)
	2019 (n, %)	1250 (21.8%)	2737 (18.4%)
*Regions in Germany*			
	Northeast (n, %)	950 (16.6%)	2749 (18.4%)
	Northwest (n, %)	1689 (29.5%)	2677 (18.0%)
	West (n, %)	1810 (31.6%)	6350 (42.6%)
	Southwest (n, %)	522 (9.1%)	1165 (7.8%)
	Southeast (n, %)	676 (11.8%)	1971 (13.2%)
	NA (n, %)	86 (1.5%)	0 (0.0%)

### Pathogen Spectrum in Dental and Oral-Maxillofacial Medicine

**FIGURE 1:**
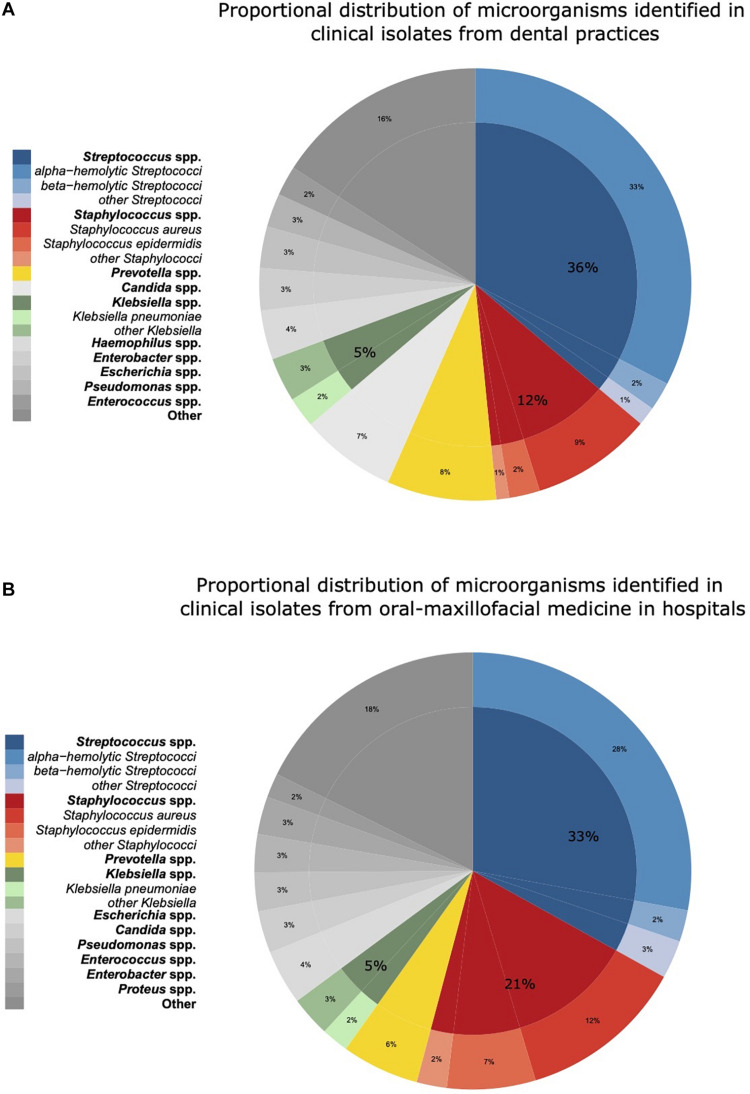
Proportional distribution of genera and species of microorganisms identified in clinical isolates from dental and oral-maxillofacial settings in Germany (2012–2019) stratified by care setting: **(A)** dental practices and **(B)** hospitals. Proportional distribution of genera and species among all clinical isolates (dental practices *n* = 5,733, hospitals *n* = 14,912) are expressed as mean proportions (%).

The proportional distributions of the pathogens identified in clinical isolates in dental and oral-maxillofacial settings are shown in Figure 1. A total of 224 different species from 73 genera were found in clinical isolates from dental practices, and 329 different species from 97 genera were identified in isolates from hospital patients.

In hospitals, *Streptococcus* spp. was identified as the most abundant pathogen (33.1%), followed by *Staphylococcus* spp. (21.1%), *Prevotella* spp. (5.7%), and *Klebsiella* spp. (5.0%). Within the *Streptococcus genus*, alpha-hemolytic *Streptococci* were most frequent in hospitals (27.9%). In contrast, *Staphylococcus* spp. was less frequently found in dental practices than in hospitals (12.3 vs. 21.1%). Notably, *Candida* spp. was more frequently identified in isolates from dental practices (8.2 vs. 3.1%). Apart from *Staphylococcus spp*. and *Candida spp*., the spectrum of pathogens was similar between isolates from hospitals and dental practices. A comprehensive overview of the pathogen identification results is provided in Supplementary Table 1. Over the course of the study period, the distribution of the major pathogen groups did not systemically change with the exception of *Staphylococcus* spp. in dental practices. The proportion of *Staphylococcus* spp. isolated among all isolated pathogens from patients treated in dental practices continuously increased from 9% in 2012–2013 to 15% in 2018–2019.

### Antimicrobial Resistance Profiles in Dental and Oral-Maxillofacial Settings

**FIGURE 2:**
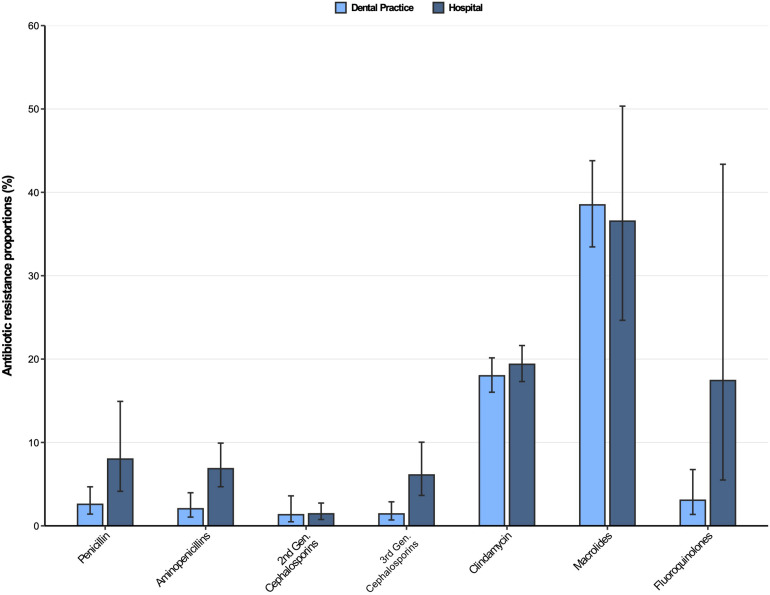
Antibiotic resistance proportions in clinical *Streptococcus* spp. isolates from dental and oral-maxillofacial settings in Germany (2012–2019) stratified by care setting: Dental practices and hospitals. Resistance proportions with corresponding 95% confidence intervals are expressed as proportions (%) of isolates tested as “resistant” among all isolates that were tested against the respective antibiotic. Total numbers of tested isolates per care setting: dental practice: penicillin: *n* = 2,016, aminopenicillins: *n* = 2,004, second-generation cephalosporins: *n* = 525, third-generation cephalosporins: *n* = 1,752, clindamycin: *n* = 2,034, macrolides: *n* = 1,130, fluoroquinolones: *n* = 456; hospitals: penicillin: *n* = 4,883, aminopenicillins: *n* = 4,099, second-generation cephalosporins: *n* = 2,496, third-generation cephalosporins: *n* = 4,491, clindamycin: *n* = 4,831, macrolides: *n* = 1,754, fluoroquinolones: *n* = 1,639.

The resistance patterns of *Streptococcus* spp. isolates from dental and oral-maxillofacial settings for various antibiotics are represented in Figure 2. In *Streptococcus* spp. isolates from hospital patients, penicillin and aminopenicillin resistance proportions were 8.0% (95%CI 4.7–14.9%) and 6.9% (95%CI 4.7–9.9%), respectively. Substantially lower penicillin and aminopenicillin resistance proportions were observed in *Streptococcus* spp. isolates from dental practices [2.6% (95%CI 1.4–4.7%) and 2.1% (95%CI 1.1–4.0%), respectively]. A multivariable analysis adjusting for some factors (Supplementary Tables 2, 3) that could influence antimicrobial resistance confirmed that *Streptococcus* spp. isolates from hospitals have a greater likelihood of exhibiting penicillin and aminopenicillin resistance than isolates from dental practices [adjusted odds ratios: 3.35 (95%CI 1.46–7.72), *p* = 0.00503 and 3.85 (95%CI 1.82–8.16), *p* < 0.001].

Resistance proportions in *Streptococcus* spp. isolates for second-generation and third-generation cephalosporins, as well as fluoroquinolones, were generally low (<7%), except for fluoroquinolone resistance proportion in hospitals [17.4% (95%CI 5.5–43.4%)]. Importantly, third-generation cephalosporins resistance proportions were higher in *Streptococcus* spp. isolates from patients treated in hospitals compared to those treated in dental practices [6.1% (95%CI 3.7–10.0%) vs. 1.4% (95%CI 0.70–2.9%)], supported by a multivariable analysis [adjusted odds ratio: 6.45 (95%CI 2.91–14.28), *p* ≤ 0.001] (Supplementary Table 4). Importantly, time trend analyses showed that third-generation as well as second-generation cephalosporin resistances in *Streptococcus* spp. isolates increased over the course of the study period [adjusted odds ratios: 1.34 (95%CI 1.02–1.76), *p* = 0.037 and 1.37 (95%CI 1.17–1.61), *p* < 0.001]. Our findings indicate that there was a relatively high resistance against clindamycin in both hospital and dental practices [19.4% (95%CI 17.3–21.6%) and 18.0% (95%CI 16.0–20.2%)]. High resistance proportions among clinical *Streptococcus* spp. isolates from hospitals and dental practices were observed for macrolides [36.6% (95%CI 24.7–50.3%) and 38.5% (95%CI 33.5–43.8%)].

**FIGURE 3:**
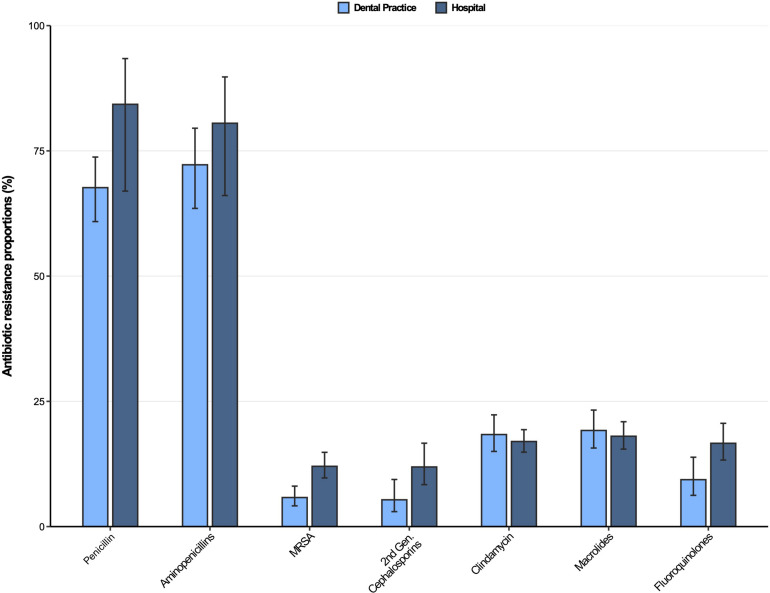
Antibiotic resistance proportions in clinical *Staphylococcus aureus* isolates from dental and oral-maxillofacial settings in Germany (2012–2019) stratified by care setting: Dental practices and hospitals. Resistance proportions with corresponding 95% confidence intervals are expressed as proportions (%) of isolates tested as “resistant” among all isolates that were tested against the respective antibiotic. Total numbers of tested isolates per care setting: dental practice: Penicillin: *n* = 470, aminopenicillins: *n* = 198, methicillin-resistant *Staphylococcus aureus*: *n* = 517, second-generation cephalosporins: *n* = 243, clindamycin: *n* = 517, macrolides: *n* = 516, fluoroquinolones: *n* = 491; hospitals: penicillin: *n* = 1,751, aminopenicillins: *n* = 657, methicillin-resistant *Staphylococcus aureus*: *n* = 1,828, second-generation cephalosporins: *n* = 1,008, clindamycin: *n* = 1,831, macrolides: *n* = 1,824, fluoroquinolones: *n* = 1,828.

Antibiotic resistance profiles for *Staphylococcus aureus* isolates from dental and oral-maxillofacial settings are displayed in Figure 3. As expected, penicillin and aminopenicillin resistance proportions were very high (>65%) in *S. aureus* isolates from hospitals and dental practices. The methicillin resistance proportion (i.e., resistance to oxacillin / flucloxacillin) among *S. aureus* isolates were significantly higher in isolates from patients treated in hospitals compared to those treated in dental practices [12.0% (95%CI 9.7–14.8%) vs. 5.8% (95%CI 4.1–8.1%)]. This finding is also confirmed by a multivariable analysis that shows that *S. aureus* isolates from hospitals had a greater likelihood to exhibit methicillin resistance compared to isolates from dental practices [adjusted odds ratio: 2.48 (95%CI 1.58–3.90), *p* < 0.001] (Supplementary Table 5). For clindamycin and macrolides, resistance proportions were relatively high (>17%) in *S. aureus* isolates from both hospitals and dental practices. Importantly, compared to isolates from hospital patients, fluoroquinolone resistance proportions were much lower in isolates from dental practices [16.6% (95%CI 13.3–20.6%) vs. 9.4% (95%CI 6.2–13.8%)], which is also supported by a multivariable analysis [adjusted odds ratio: 2.28 (95%CI 1.56–3.31), *p* < 0.001] (Supplementary Table 6). Time trend analyses revealed that fluoroquinolone resistances in *S. aureus* isolates decreased between 2012 and 2019 [adjusted odds ratio: 0.91 (95%CI 0.86–0.97), *p* = 0.004].

**FIGURE 4:**
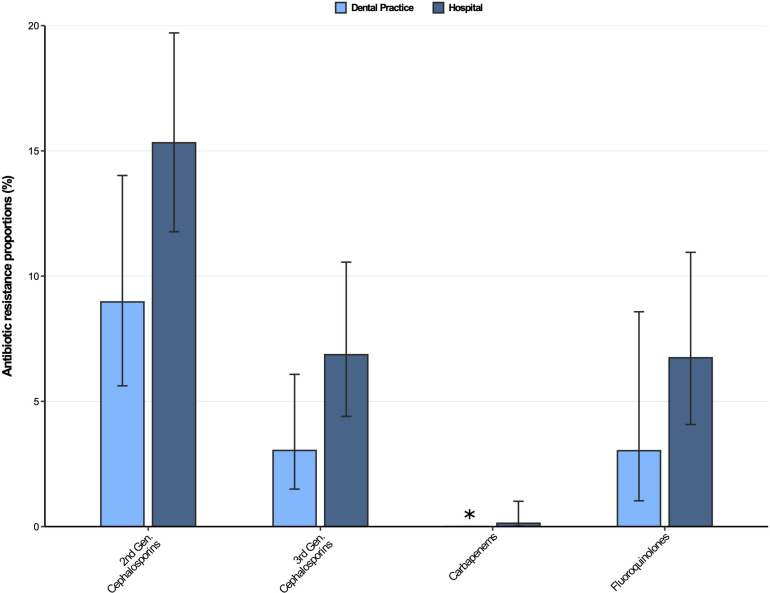
Antibiotic resistance proportions in clinical *Klebsiella* spp. isolates from dental and oral-maxillofacial settings in Germany (2012–2019) stratified by care setting: Dental practices and hospitals. Resistance proportions with corresponding 95% confidence intervals are expressed as proportions (%) of isolates tested as “resistant” among all isolates that were tested against the respective antibiotic. Total number of tested isolates per care setting: dental practice: second-generation cephalosporins: *n* = 223, third-generation cephalosporins: *n* = 296, carbapenems: *n* = 298, fluoroquinolones: *n* = 264; hospitals: second-generation cephalosporins: *n* = 633, third-generation cephalosporins: *n* = 743, carbapenems: *n* = 744, fluoroquinolones: *n* = 727. ^∗^ All isolates were tested sensible.

The antibiotic resistance proportions for *Klebsiella* spp. isolates from dental and oral-maxillofacial settings are displayed in Figure 4. Moderately high resistance proportions (9–16%) were found for second-generation cephalosporins, while low resistance proportions (<8%) were observed for third-generation cephalosporins, as well as fluoroquinolones in isolates from both hospitals and dental practices. However, an increase of fluoroquinolone resistance in *Klebsiella* spp. isolates was observed over the course of the study period [adjusted odds ratio: 1.14 (95%CI 1.02–1.27), *p* = 0.026]. Importantly, carbapenem resistance among *Klebsiella* spp. isolates was very rare (dental practices: 0%, hospitals 0.13%).

## Discussion

This study analyzed the spectrum of pathogens and antibiotic resistance profiles in both the outpatient (i.e., dental practices) and hospital sector of dentistry using data from the German *Antibiotic-Resistance-Surveillance* database. Our study identified 360 individual species from more than 106 different genera in clinical samples from patients with dental and oral-maxillofacial infections, reflecting the highly diverse microbiome of the oral cavity. Oral-maxillofacial infections are often characterized by a mixed growth of anaerobic and aerobic bacteria (Bahl et al., 2014). Although some genera / species were exclusively isolated in outpatient dental practices or hospitals, in more than 85% of the included isolates, genera / species were identified that were found in both healthcare settings. Our results show that the most frequently found pathogens were gram-positive cocci, especially *Streptococcus* spp. and *Staphylococcus* spp. Alpha-hemolytic *Streptococci*, which also include the viridans group of *Streptococci* (Facklam, 2002), are the largest individual group of pathogens to be detected in clinical samples from the oral cavity. In line with our findings, studies from other parts of the world also found that *Streptococci*, especially species from the viridans group, are frequent causes of odontogenic infections (Farmahan et al., 2014; Al-Nawas and Karbach, 2017; Heim et al., 2017; Haque et al., 2019). *Streptococci* are commensals typically found in the oral cavity, but they are also commonly found in intestinal tract and genital area in healthy individuals. However, under conditions where the microbiota balance is disrupted, *Streptococci* can cause infections, including dental and oral-maxillofacial infections.

The second most frequent pathogen group found in this study were *Staphylococci*. Until recently, the *Staphylococcus* species were not considered member of the oral flora. Heim et al. (2017) investigated head and neck space infections of odontogenic origin in 2018 and found that *Staphylococcus* species are one of the four most commonly isolated bacteria. Smith and colleagues (Smith et al., 2001) noted that the *Staphylococcus* species are a more frequent colonizer of the oral cavity than previously thought. Nevertheless, its role as a transient member of the oral microbiome or a possible pathogen is not fully understood yet (Koukos et al., 2015).

Interestingly, while the *Candida* spp. were not prominently encountered in clinical samples from hospital patients with odontogenic infections, these fungal pathogens were found in 6% of all clinical samples from patients treated in outpatient dental practices, underlining their importance in community settings. Although oral candidiasis is not harmful to otherwise healthy people, it can be more severe and difficult to control in people who are immune-compromised (Ostrosky-Zeichner and Pappas, 2006).

Our analyses of antibiotic resistance profiles shows that clinical *Streptococcus* spp. and *S. aureus* isolates from both hospital and dental practices show relatively high proportions of clindamycin resistance (17–19%). This finding is of clinical importance, since clindamycin is one of the most frequently prescribed antibiotics by dental practitioners in Germany (Halling et al., 2017). Notably, the German guidelines on odontogenic infections recommends penicillin and amoxicillin for empiric antibiotic therapy, while clindamycin is only recommended in cases of penicillin allergy (Al-Nawas and Karbach, 2017). In line with our findings, Heim et al. (2017) and Poeschl et al. (2010) reported similar clindamycin resistance rates for *Streptococci* and *S. aureus* in Germany and Austria. It is encouraging that resistance proportions against the recommended first-line antibiotics penicillin and aminopenicillins (including amoxicillin) are very low (<3%) in clinical *Streptococcus* spp. isolates from dental practices.

Although significantly higher resistance rates were found in *Streptococcus* spp. isolates from hospitals, penicillin and aminopenicillin resistance proportions remain moderate (∼7–8%), but continuous efforts in antibiotic stewardship are needed to preserve the clinical effectiveness of these antibiotics. As expected, *S. aureus* shows very high resistance proportions against penicillin and aminopenicillin (>65%). Together with its relatively high clindamycin and macrolide resistance proportions (>17%), treatment options are very limited, which is concerning since our results indicate that *S. aureus* is frequently found in odontogenic infections. Importantly, in our study on dental and oral-maxillofacial infections, the proportion of β-lactam penicillinase resistance (i.e., methicillin resistance) in *S. aureus* isolates from dental practices (6%) and hospitals (12%) are similar to MRSA proportions observed in 2018 in other clinical samples from outpatient and hospital settings in Germany. In general, there has been a decrease in the MRSA proportion in all *S. aureus* isolates from all specimen materials from 2010 to 2018. In hospitals, the MRSA proportion declined from 23.8 to 13.3%, and in the outpatient sector from 13.0 to 7.7% (Layer et al., 2019).

Both *Streptococcus* spp. and *S. aureus* show relatively high resistance proportions (>18%) against macrolides in hospitals as well as in dental practices. These findings are somewhat contrary to other studies, which only found resistance proportions up to 13% in the hospital setting (Poeschl et al., 2010) for *Streptococcus* species.

Although our dataset on antibiotic resistance profiles is limited for *Klebsiella* spp. isolates, our data indicate moderate to low resistance proportions for cephalosporins and fluoroquinolones. Importantly, only one carbapenem-resistant isolate was found in a hospital sample, which is in line with findings from Koppe et al. (2018), who found that less than 1% of all *Klebsiella* spp. isolates from German hospitals were non-susceptible to carbapenems. In line with these results, low carbapenem resistance rates were also found for other Gram-negative bacteria in Germany, such as *Acinetobacter baumannii* (Said et al., 2021) and *E. coli* (ECDC, 2020).

Our results demonstrate that frequent Gram-positive pathogens isolated from clinical odontogenic samples show substantial antibiotic resistance against important antibiotics. Moreover, although resistances against third and secondary cephalosporins in *Streptococcus* spp. were relatively low, it is worrying that resistances against these antibiotic classes increased over the study period, which underlines the importance of continuous efforts in antibiotic stewardship. In Germany, about 10% of all antibiotics are prescribed by dentists (Halling et al., 2017). It is estimated that approximately one-third of all outpatient antibiotic prescriptions are unnecessary (Fleming-Dutra et al., 2016) and thereby contribute to the development of antibiotic resistance. The potential overuse of antibiotics (e.g., in antibiotic prophylaxis) is rarely addressed in dentistry, but a recent study by Löffler and Böhmer (2017) showed that a combination of audit and feedback and education on antibiotics could help as an intervention in hospital dental care and outpatient dental settings.

When interpreting our data, it is important to consider that microbiological sampling is not routinely performed in the management of patients with clinical infections in outpatient dental practices. It is likely that the included isolates from dental practices represent infections episodes with higher severity, chronic progressions and/or complications, such as treatment failure in first-line antibiotic therapy. Therefore, the observed pathogen spectrum and associated antibiotic resistances may not be generalizable for all clinical infections treated in dental practices. In addition, information on any antibiotic treatment before sampling is lacking, which can also bias the data.

### Strengths and Limitations

This study is based on data from the ARS database, which is the largest and most representative surveillance system for pathogen identifications and antibiotic resistance in Germany (Noll et al., 2012). To our knowledge, this multicenter study is the most comprehensive study on the spectrum of pathogens and antibiotic resistance profiles in Europe, and includes more than 20,700 clinical isolates from more than 12,400 patients with dental and oral-maxillofacial infections. In contrast to many previous studies, we included data from patients treated in hospital and dental practices.

However, it is also important to consider the limitations of the study. Firstly, participation in ARS is voluntary and therefore laboratories and hospitals are not evenly distributed in Germany, which may limit the representativeness of the data. However, isolates were from all major regions in Germany, without disproportionate under-representation in any particular region.

Secondly, underlying diagnoses are not collected in ARS, so we can only assume that clinical specimens represent infectious diseases. We excluded all isolates labeled as screening samples, but it is possible that some of the analyzed isolates actually represent screening samples that were not assigned as such by the hospital or laboratory. Importantly, the identified pathogens in the clinical samples may not be the actual infectious agents, but may represent commensals that “contaminated” the microbiological samples drawn from the actual infection site. In addition, about 50% of the species of the oral flora cannot be grown *in vitro* and therefore cannot be detected in standard microbiological analyses and their potential role in infectious disease remains unknown (Siqueira and Rôças, 2013).

Importantly, microbiological sampling procedures (i.e., aerobic or aerobic sampling) also impact the observed the pathogen spectrum. Although oral-maxillofacial infections are often associated with a mixed growth of anaerobic and aerobic bacteria, anaerobic bacteria are largely absent in our data set, with the exception of *Prevotella* spp., which might be explained by aerobic sampling methods in routine diagnostics. No information on the microbiological sampling method is available in the ARS database.

### Conclusion

Our study shows that dental and oral-maxillofacial infections in Germany are associated with a wide range of different pathogens, and that *Streptococci* (especially alpha-hemolytic *Streptococci*) and *Staphylococci* (especially *S. aureus*) are the most frequently identified pathogens in hospitals and dental practices. Both *Streptococcus* spp. and *S. aureus* show substantial resistance against important antibiotics, which calls for strengthened efforts in antibiotic stewardship and infection prevention and control measures in both hospitals and dental practices.

## Data Availability Statement

The raw data supporting the conclusions of this article will be made available by the authors, without undue reservation.

## Author Contributions

AM, AR, TE, and RM were responsible for conceptualization of the study and formulation of the research goals and aims. AM, NW, and RM developed the statistical methodology and models. IN and MF established and maintain the ARS database and continuously validate the data. AM and RM performed the statistical analysis. AM, AR, BA-N, and RM wrote the original draft. All authors reviewed, commented the draft and gave input on editing, read, and approved the final manuscript.

## Conflict of Interest

The authors declare that the research was conducted in the absence of any commercial or financial relationships that could be construed as a potential conflict of interest.
