# Supplementary material for: Antimicrobial Resistance and the Spectrum of Pathogens in Dental and Oral-Maxillofacial Infections in Hospitals and Dental Practices in Germany
Source: Front Microbiol. 2021 Jun 2;12:676108. doi: 10.3389/fmicb.2021.676108 (PMC8206268; doi:10.3389/fmicb.2021.676108)
Supplement: Supplementary file 1 [file Data_Sheet_1.docx]

**Supplementary Material**

**Antimicrobial resistance and the spectrum of pathogens in dental and oral-maxillofacial infections in hospitals and dental practices in Germany**

**Annika Meinen^1,*^, Annicka Reuss^1^, Niklas Willrich^1^, Marcel Feig^1^, Ines Noll^1^, Tim Eckmanns^1^, Bilal Al-Nawas^2^, Robby Markwart^1,3^**

^1^ Robert Koch Institute, Unit 37: Nosocomial Infections, Surveillance of Antimicrobial Resistance and Consumption, Seestrasse 10, D-13353 Berlin, Germany

^2^ University Medical Centre of the Johannes Gutenberg-University Mainz, Department of Oral and Maxillofacial Surgery, Plastic Surgery, Langenbeckstrasse 1, D-55131 Mainz, Germany

^3^ Jena University Hospital, Institute of General Practice and Family Medicine, Bachstrasse 18, D-07743 Jena, Germany

*** Correspondence:**Annika Meinen
meinenan@rki.de

**Supplementary Table 1.** Pathogen identification results from patients with dental and oral-maxillofacial infections.

|  | **Hospitals** | | **Outpatient dental practices** | |
| --- | --- | --- | --- | --- |
| ***Streptococcus* spp.** | | | | |
| **Pathogen identification results** | **Number of isolates** | **Proportion among all *Streptococcus* spp. isolates (n = 4930)** | **Number of isolates** | **Proportion among all *Streptococcus* spp. isolates (n = 2072)** |
| Viridans streptococci | 1360 | 27.6% | 274 | 13.2% |
| *S. constellatus* | 651 | 13.2% | 106 | 5.1% |
| *S. anginosus* | 591 | 12.0% | 98 | 4.7% |
| *Streptococcus* spp. | 399 | 8.1% | 61 | 2.9% |
| *S. mitis* | 360 | 7.3% | 520 | 25.1% |
| *S. oralis* | 294 | 6.0% | 555 | 26.8% |
| *S. indermedius* | 289 | 5.9% | 41 | 2.0% |
| *S. parasinguinis* | 191 | 3.9% | 68 | 3.3% |
| *S. salivarius* | 128 | 2.6% | 88 | 4.3% |
| *S. sanguinis* | 108 | 2.2% | 38 | 1,8% |
| Other | 559 | 11.3% | 223 | 10.8% |
| ***Staphylococcus* spp.** | | | | |
| **Pathogen identification results** | **Number of isolates** | **Proportion among all *Staphylococcus* spp. isolates (n = 3144)** | **Number of isolates** | **Proportion among all *Staphylococcus* spp. isolates (n = 705)** |
| *S. aureus* | 1834 | 58.3% | 518 | 73.5% |
| *S. epidermidis* | 977 | 31.1% | 132 | 18.7% |
| *S. capitis* | 87 | 2.8% | 5 | 0.7% |
| Coagulase-negative staphylococci | 73 | 2.3% | 27 | 3.8% |
| *S. hominis* | 56 | 1.8% | 4 | 0.6% |
| *S. lugdunensis* | 41 | 1.3% | 7 | 1.0% |
| *S. haemolyticus* | 31 | 1.0% | 2 | 0.3% |
| *S. warneri* | 15 | 0.5% | 4 | 0.6% |
| *S. caprae* | 5 | 0.2% | - | - |
| Other | 25 | 0.8% | 6 | 0.9% |
|  | **Hospitals** | | **Outpatient dental practices** | |
| ***Prevotella* spp.** | | | | |
| **Pathogen identification results** | **Number of isolates** | **Proportion among all *Prevotella* spp. isolates (n = 854)** | **Number of isolates** | **Proportion among all *Prevotella* spp. isolates (n = 469)** |
| *P. melaninogenica* | 187 | 21.9% | 180 | 38.8% |
| *Prevotella* spp. | 184 | 21.6% | 79 | 16.8% |
| *P. buccae* | 166 | 19.4% | 54 | 11.5% |
| *P. denticola* | 126 | 14.8% | 47 | 10.0% |
| *P. intermedia* | 84 | 9.8% | 39 | 8.3% |
| *P. oralis* | 43 | 5.0% | 28 | 6.0% |
| *P. oris* | 30 | 3.5% | 12 | 2.6% |
| *P. bivia* | 11 | 1.3% | 11 | 2.4% |
| *P. loeschii* | 8 | 0.9% | 8 | 1.7% |
| *P. disiens* | 5 | 0.6% | - | - |
| Other | 10 | 1.2% | 13 | 2.8% |
| ***Klebsiells* spp.** | | | | |
| **Pathogen identification results** | **Number of isolates** | **Proportion among all *Klebsiella* spp. isolates (n = 745)** | **Number of isolates** | **Proportion among all *Klebsiella* spp. isolates (n = 321)** |
| *K. pneumoniae* | 301 | 40.4% | 131 | 40.8% |
| *K. oxytoca* | 291 | 39.1% | 154 | 48.0% |
| *K. aerogenes* | 80 | 10.7% | 23 | 7.2% |
| *Klebsiella* spp. | 69 | 9.3% | 11 | 3.4% |
| *K. varicola* | 3 | 9.4% | - | - |
| *K. ozaenae* | 1 | 0.1% | 2 | 0.6% |

**Supplementary Table 2.** Logistic regression analysis of factors associated with penicillin resistance in *Streptococcus spp.* isolates in Germany (n=6899).

|  |  | | ***Multivariable analysis*** | | |
| --- | --- | --- | --- | --- | --- |
|  |  | | ***OR*** | ***(95% CI)*** | ***p-value*** |
| ***Year of sampling (per 1 year increase)*** | | |  |  |  |
|  | 2012-2019 | | 0.98 | (0.87-1.11) | 0.760 |
| ***Care setting type*** | | | | | |
|  | Dental practices | 1 | | - | - |
|  | Hospitals | 3.35 | | (1.46-7.72) | 0.00503 |
| ***Patient gender*** | |  | |  |  |
|  | Male | 1 | | - | - |
|  | Female | 1.09 | | (0.79-1.49) | 0.597 |
|  | Unknown | 1.21 | | (0.50-2.91) | 0.677 |
| ***German region*** | |  | |  |  |
|  | Northeast | 1 | | - | - |
|  | Southeast | 0.99 | | (0.039-0.25) | <0.001 |
|  | Southwest | 0.40 | | (0.20-0.82) | 0.0127 |
|  | West | 0.25 | | (0.12-0.53) | <0.001 |
|  | Northwest | 0.26 | | (0.14-0.49) | <0.001 |
|  | Unknown | <0.001 | | (<0.001) | <0.001 |
| ***Patient age*** | |  | |  |  |
|  | ≤19 years | 1 | | - | - |
|  | 20-39 years | 1.00 | | (0.60-1.68) | 0.998 |
|  | 40-64 years | 0.91 | | (0.56-1.49) | 0.704 |
|  | ≥65 years | 0.85 | | (0.49-1.48) | 0.569 |
|  |  |  | |  |  |

**Supplementary Table 3.** Logistic regression analysis of factors associated with aminopenicillin resistance in *Streptococcus spp.* isolates in Germany (n=6103).

|  |  | | ***Multivariable analysis*** | | |
| --- | --- | --- | --- | --- | --- |
|  |  | | ***OR*** | ***(95% CI)*** | ***p-value*** |
| ***Year of sampling (per 1 year increase)*** | | |  |  |  |
|  | 2012-2019 | | 1.01 | (0.92-1.11) | 0.771 |
| ***Care setting type*** | | | | | |
|  | Dental practices | 1 | | - | - |
|  | Hospitals | 3.85 | | (1.82-8.16) | <0.001 |
| ***Patient gender*** | |  | |  |  |
|  | Male | 1 | | - | - |
|  | Female | 1.10 | | (0.92-1.32) | 0.275 |
|  | Unknown | 1.29 | | (0.59-2.83) | 0.523 |
| ***German region*** | |  | |  |  |
|  | Northeast | 1 | | - | - |
|  | Southeast | 0.17 | | (0.059-0.49) | 0.00135 |
|  | Southwest | 0.63 | | (0.35-1.11) | 0.113 |
|  | West | 0.45 | | (0.29-0.69) | <0.001 |
|  | Northwest | 0.74 | | (0.44-1.27) | 0.279 |
|  | Unknown | <0.001 | | (<0.001) | <0.001 |
| ***Patient age*** | |  | |  |  |
|  | ≤19 years | 1 | | - | - |
|  | 20-39 years | 0.64 | | (0.34-1.21) | 0.170 |
|  | 40-64 years | 0.68 | | (0.39-1.18) | 0.171 |
|  | ≥65 years | 0.69 | | (0.37-1.30) | 0.253 |
|  |  |  | |  |  |

**Supplementary Table 4.** Logistic regression analysis of factors associated with third-generation cephalosporin resistance in *Streptococcus spp.* isolates in Germany (n=6243).

|  |  | | ***Multivariable analysis*** | | |
| --- | --- | --- | --- | --- | --- |
|  |  | | ***OR*** | ***(95% CI)*** | ***p-value*** |
| ***Year of sampling (per 1 year increase)*** | | |  |  |  |
|  | 2012-2019 | | 1.34 | (1.02-1.76) | 0.0365 |
| ***Care setting type*** | | | | | |
|  | Dental practice | 1 | | - | - |
|  | Hospitals | 6.45 | | (2.91-14.28) | <0.001 |
| ***Patient gender*** | |  | |  |  |
|  | Male | 1 | | - | - |
|  | Female | 0.99 | | (0.84-1.15) | 0.872 |
|  | Unknown | 1.02 | | (0.43-2.39) | 0.968 |
| ***German region*** | |  | |  |  |
|  | Northeast | 1 | | - | - |
|  | Southeast | 0.24 | | (0.048-1.19) | 0.0822 |
|  | Southwest | 0.74 | | (0.26-2.06) | 0.565 |
|  | West | 0.40 | | (0.18-0.886) | 0.0254 |
|  | Northwest | 1.49 | | (0.78-2.85) | 0.226 |
|  | Unknown | <0.001 | | (<0.001) | <0.001 |
| ***Patient age*** | |  | |  |  |
|  | ≤19 years | 1 | | - | - |
|  | 20-39 years | 0.92 | | (0.48-1.76) | 0.805 |
|  | 40-64 years | 0.69 | | (0.39-1.22) | 0.204 |
|  | ≥65 years | 0.76 | | (0.40-1.44) | 0.405 |
|  |  |  | |  |  |

**Supplementary Table 5.** Logistic regression analysis of factors associated with MRSA resistance in *Staphylococcus aureus* isolates in Germany (n=2345).

|  |  | | ***Multivariable analysis*** | | |
| --- | --- | --- | --- | --- | --- |
|  |  | | ***OR*** | ***(95% CI)*** | ***p-value*** |
| ***Year of sampling (per 1 year increase)*** | | |  |  |  |
|  | 2012-2019 | | 0.93 | (0.84-1.04) | 0.186 |
| ***Care setting type*** | | | | | |
|  | Dental practices | 1 | | - | - |
|  | Hospitals | 2.48 | | (1.58-3.90) | <0.001 |
| ***Patient gender*** | |  | |  |  |
|  | Male | 1 | | - | - |
|  | Female | 0.66 | | (0.43-0.99) | 0.0479 |
|  | Unknown | 1.16 | | (0.77-1.76) | 0.476 |
| ***German region*** | |  | |  |  |
|  | Northeast | 1 | | - | - |
|  | Southeast | 1.85 | | (1.16-2.95) | 0.0122 |
|  | Southwest | 0.81 | | (0.56-1.18) | 0.273 |
|  | West | 0.67 | | (0.46-0.99) | 0.0449 |
|  | Northwest | 1.00 | | (0.72-1.39) | 0.997 |
|  | Unknown | 4.94 | | (1.12-21.85) | 0.0370 |
| ***Patient age*** | |  | |  |  |
|  | ≤19 years | 1 | | - | - |
|  | 20-39 years | 0.99 | | (0.37-2.66) | 0.983 |
|  | 40-64 years | 0.77 | | (0.33-1.83) | 0.557 |
|  | ≥65 years | 0.99 | | (0.44-2.26) | 0.990 |
|  |  |  | |  |  |

**Supplementary Table 6.** Logistic regression analysis of factors associated with fluoroquinolone resistance in *Staphylococcus aureus* isolates in Germany (n=2319).

|  |  | | ***Multivariable analysis*** | | |
| --- | --- | --- | --- | --- | --- |
|  |  | | ***OR*** | ***(95% CI)*** | ***p-value*** |
| ***Year of sampling (per 1 year increase)*** | | |  |  |  |
|  | 2012-2019 | | 0.91 | (0.86-0.97) | 0.00364 |
| ***Care setting type*** | | | | | |
|  | Dental practices | 1 | | - | - |
|  | Hospitals | 2.28 | | (1.56-3.31) | <0.001 |
| ***Patient gender*** | |  | |  |  |
|  | Male | 1 | | - | - |
|  | Female | 0.90 | | (0.60-1.37) | 0.638 |
|  | Unknown | 0.93 | | (0.63-1.37) | 0.719 |
| ***German region*** | |  | |  |  |
|  | Northeast | 1 | | - | - |
|  | Southeast | 3.49 | | (2.32-5.25) | <0.001 |
|  | Southwest | 0.77 | | (0.56-1.07) | 0.125 |
|  | West | 1.12 | | (0.77-1.63) | 0.560 |
|  | Northwest | 1.55 | | (1.18-2.05) | 0.0230 |
|  | Unknown | 4.04 | | (1.24-13.15) | 0.0218 |
| ***Patient age*** | |  | |  |  |
|  | <19 years | 1 | | - | - |
|  | 20-39 years | 0.66 | | (0.25-1.74) | 0.404 |
|  | 40-64 years | 1.24 | | (0.60-2.55) | 0.562 |
|  | ≥65 years | 2.03 | | (0.94-4.35) | 0.0721 |
|  |  |  | |  |  |
